# Removal of Congo Red Dye from Aqueous Solution via Natural Seeds Integrated with Zinc Oxide-Doped Manganese Ferrite

**DOI:** 10.3390/nano16120775

**Published:** 2026-06-19

**Authors:** Elham A. Alzahrani, Ghaida H. Munshi, Abeer Mohammed AL-Balawi, Salwa D. Al-Malwi, Naha Meslet Alsebaii, Khloud Saeed Al-Thubaiti, Sumbul Hafeez, Seungdae Oh

**Affiliations:** 1 Department of Chemistry, College of Science, University of Ha’il, Ha’il 81451, Saudi Arabia; elhama.alzahrani@gmail.com; 2 Department of Chemistry, College of Science, University of Jeddah, Jeddah 21959, Saudi Arabia; gmunshi@uj.edu.sa; 3 Department of Chemistry, University Duba College, University of Tabuk, Tabuk 71491, Saudi Arabia; ab_albalawi@ut.edu.sa; 4 Department of Chemistry, College of Science, Northern Border University, Arar 91431, Saudi Arabia; salwa.almaalawi@nbu.edu.sa; 5 Department of Chemistry, Faculty of Science, King Abdulaziz University, Jeddah 21589, Saudi Arabia; nalsebaii@kau.edu.sa (N.M.A.); halh_4488@hotmail.com (K.S.A.-T.); 6 Department of Civil and Environmental Engineering, Villanova University, Lancaster, PA 19085, USA; 7 Department of Civil Engineering, College of Engineering, Kyung Hee University, Yongin 17104, Republic of Korea

**Keywords:** black cumin, ZnO, MnFe_2_O_4_, Congo red, adsorption, water treatment

## Abstract

This study reports the preparation of a nanocomposite using a black cumin surface as a carbon framework on which zinc oxide-doped manganese ferrite nanoparticles were deposited and grown. A simple precipitation method was used to prepare the nanocomposite. The resulting composite was characterized using various characterization analyses such as FTIR, XRD, EDX, SEM, TEM, and TGA. The composite surface was highly conformed with functional groups, and the nanocomposite was formed due to electrostatic and non-electrostatic interactions between the carbon framework and the nanoparticles. X-ray analysis revealed a crystalline structure with crystal sizes up to 45 nm. Microscopic images revealed the surface morphology, confirming the irregular distribution of particles within the composite. The resulting composite material was used for adsorption application. The composite material was tested for the removal of Congo red dye from water. It was found that under optimal conditions, a dose of 2 g per liter of absorbent removed nearly 100% of dye from a 10 mL volume of 10 mg per liter Congo red solution within 90 min and 7 pH. A monolayer adsorption was confirmed by the isotherm analysis. The monolayer adsorption capacity for the present study was ~13.0 mg per gram. The adsorption kinetics suggested the fitting of pseudo-second order. Based on the findings, it was concluded that the chemical mechanism was responsible for the present adsorption process. The regeneration study demonstrates the stability of current adsorbent up to two cycles only. This nanocomposite is the first of its kind which promotes the creation of nanocomposites in the future by using natural materials and reduces the dependency on activated carbon.

## 1. Introduction

The problem of dye pollution in water has increased with industrial activity, putting human life at risk [1]. Dyes are released from various industrial processes and contaminate water [1]. Dyes are complex organic structures that produce benzidine metabolites, which are responsible for toxicity [2]. Dyes are basically composed of azo groups [2]. Sunlight penetration decreases due to high concentration of dyes, affecting flora and fauna in the water [3]. Dye produces various toxicities in the human body as well [1]. Due to the highly toxic nature of dyes, it is extremely important to remove them from water, for which various methods are used, such as physical, chemical, and biological methods [4]). The adsorption method is considered the best method. In the adsorption method, a pollutant, called an adsorbate, is adsorbed by a solid surface acting as an adsorbent through physical or chemical bonds. The adsorption method is effective for the treatment and removal of pollutants present in air and water environments [5,6,7]).

The adsorption efficiency of an adsorbent depends on the surface area and the functional groups present on it. That is, the adsorption efficiency can be increased or decreased by changing the surface properties of the adsorbent [8,9].

The most prominent adsorbents are biochar and activated carbon, which are highly porous and derived from natural materials [10]. Biochar/Activated carbon has been used to remove a variety of pollutants [11,12,13]. To advance the adsorption properties of activated carbon/biochar, they have been modified with nanomaterials, which significantly enhance the adsorption activity of modified activated carbon compared to unmodified activated carbon and also increase the adsorption efficiency [14]. For example, by combining activated carbon with magnetic nanoparticles, activated carbon can be used for magnet-responsive separation [15]. Similarly, by adding a photocatalyst such as titanium oxide to activated carbon, activated carbon can exhibit both adsorption and photocatalytic degradation properties [16]. Considering the properties of these prepared activated carbon-based nanocomposites, many modified activated carbon nanocomposites have been used in dye adsorption. However, it is important to note that these activated carbons, or biochars, are produced by burning carbon sources in a furnace at very high temperatures, requiring a high-temperature heating, which consumes significant energy and electricity. Furthermore, chemicals such as sulfuric acid, phosphoric acid, and hydrochloric acid are also used to activate them [17]. These factors increase the cost of activated carbon. Furthermore, secondary pollutants are also produced during the production of activated carbon, which reduces its applicability. Although starch [18], chitosan [19], cellulose [20], and other activated carbon alternatives such as graphene oxide have also been used [21], demonstrating excellent results, given the demand for adsorbents and high efficiency, several other alternatives to activated carbon are being explored.

New research has been carried out in which nanocomposites were formed by directly depositing nanomaterials on plant seeds or leaves and such materials showed a better adsorption efficiency as well as many advanced properties [22]). For example, iron oxide was deposited on guava leaf, due to which guava leaf showed a magnetic response, and was used in the adsorption of Methylene Blue (MB) [23]. A composite was formed by depositing manganese oxide on the surface of black cumin (BC), which also displayed an adsorption property along with antibacterial and photocatalytic properties [24]. Because these nanocomposites are made by depositing them on natural substances which are easily and cheaply available, due to the presence of phytochemicals in them they also have many medicinal properties which help in making the prepared nanocomposites non-toxic, cheap and more porous adsorbent [25].

Given the demand for these nanocomposites and their features, this study also developed a natural carbon-based nanocomposite material using black cumin (BC) as a carbon framework. BC, a readily available herbaceous plant, contains a variety of highly medically active phytochemicals [25]. With the help of its carbon framework, various nanomaterials have been deposited on its surface to form nanocomposites [25]. The BC surface is a cellulosic and protein-rich surface containing multiple functional groups. These functional groups help in adsorbing and depositing metal ions on its surface, which are then precipitated with the help of a precipitating agent such as base [25]. Additionally, BC is an oil-rich natural substance that readily leaves extracts in water. During the preparation of nanocomposites, these extracts and oils help in the reduction of metals on their surfaces and also act as functionalization and capping agents. The size of the NPs deposited on the composites is very small and the resulting NMs appears to be stable [26,27].

Considering this theoretical hypothesis, the objective of the present study is to prepare a nanocomposite by depositing zinc oxide (ZnO) and manganese ferrite (MnFe_2_O_4_) on a BC surface via a simple co-precipitation method. The successful incorporation of MnFe_2_O_4_ on BC surface has been demonstrated previously [28]. The novelty of this study is to incorporate ZnO-integrated MnFe_2_O_4_ onto the BC seeds. The addition of ZnO in this study may increase the electrical conductivity and photocatalytic activity of the developed composite, along with its mechanical strength, which is crucial for wastewater treatment.

The further objective of the present study is to demonstrate the adsorption activity of the prepared nanocomposite via the kinetics, isotherm, and thermodynamics parameters for CR dye removal.

## 2. Materials and Method

### 2.1. Materials

Zinc (II) nitrate hexahydrate, Zn(NO_3_)_2_ (molecular weight = 297.49 g/mol; purity 98%), ferric (III) chloride, FeCl_3_ (molecular weight = 162.20 g/mol; purity 99%), manganese (II) sulfate monohydrate, MnSO_4_·H_2_O (molecular weight = 169.20 g/mol; purity 98%), sodium hydroxide, NaOH (molecular weight = 40 g/mol; purity 99%), and hydrochloric acid, HCl (molecular weight = 36.46 g/mol; purity 37%) were procured from Sigma Aldrich, Darmstadt, Germany. Congo red dye, C_32_H_22_N_6_Na_2_O_6_S_2_ (Molecular weight = 696.65 g/mol), was procured from Sigma Aldrich, Darmstadt, Germany. Black cumin seeds were purchased from the local market, Hai’l City, Saudi Arabia. Seeds were washed several times with double-distilled water, dried, and ground into the powder form.

### 2.2. Preparation of Stock Solution of Dye

Dye stock solution was prepared by dissolving 1 g of dye in 1 L of distilled water, which was then diluted as required.

### 2.3. Preparation of Nanocomposite

**Scheme 1:**
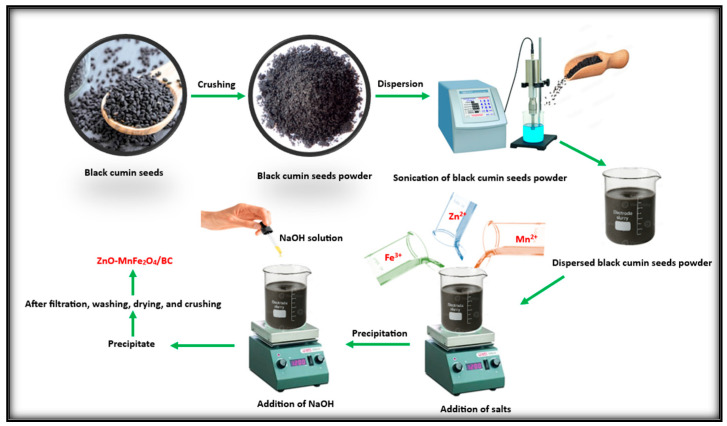
Schematic diagram of stepwise preparation of ZnO-MnFe_2_O_4_/BC.

A simple co-precipitation method was used to prepare the ZnO-MnFe_2_O_4_/BC nanocomposite [29]. During the preparation, plant extracts were also released at a given temperature (i.e., 60 °C), which also helps in bioprecipitation [26]. Briefly, first, 1 g of BC seed powder was dispersed in 100 mL of water using a Sonicator. After that, 100 mL of each of the 0.1 M, 0.05 M, and 0.05 M solutions of iron salt, FeCl_3_ (1.622 g), manganese salt, MnSO_4_·H_2_O (0.846 g), and zinc salts, Zn(NO_3_)_2_ (1.487 g), were added, respectively, to the above dispersed BC seed aqueous mixture under the continuous stirring as shown in Scheme 1. Then the solution mixture temperature was increased up to 65 °C. The solution mixture was further stirred (750 rpm) for half an hour, at 65 °C. After half an hour, 8 M of NaOH was added dropwise to the above solution mixture and the pH was increased to around 10 and maintained, which gradually started the precipitate formation. Then the reaction was run for a further 15 min until complete precipitation was achieved. The blackish-brown precipitate obtained was filtered and washed with distilled water several times, and dried. The prepared sample was dried in a hot air oven at 80 °C for 24 h. The sample was collected and stored for characterization and application.

### 2.4. Characterization and Instrumentation Analysis

Various spectroscopic and microscopic techniques were used to perform the nanocomposite characterization. For instance: Fourier transform infrared spectroscopy (FTIR) was used to determine functional groups and interaction between the carbon framework and nanoparticles, for which an FTIR spectrometer, Perkin Elmer, Waltham, MA, USA was used in the mid IR range 400–4000 cm^−1^ (using KBr pellets as a reference). To determine the crystal size and crystal structure, an X-ray diffractometer, SMARTLAB, Rigaku, Tokyo, Japan, (using Cu Kα radiation λ = 1.54 Å (40 kV, 40 mA) over the range of 10–80° (2θ)) was used. Microscopic analysis, which reveals surface and crystal morphology, was carried out using the Field Emission Scanning Electron Microscope (FESEM)-JSM-7610FPlus, JEOL, Akishima, Tokyo, Japan, and the Transmittance Electron Microscope (TEM)-F200XG2, Thermo Fisher Scientific Inc., Waltham, MA, USA. Thermogravimetric analysis (TGA) was carried out to assess the thermal stability of the nanocomposite under the flow of nitrogen gas at 20 mL min^−1^ rate using TGA 3+ with a Small Furnace (SF) instrument, METTLER TOLEDO, Greifensee, Switzerland. The TGA curve was recorded between 30 °C and 900 °C by heating at rate of 10 °C min^−1^.

### 2.5. Batch Adsorption Experiment

A batch adsorption experiment was conducted to investigate the dye removal performance of ZnO-MnFe_2_O_4_/BC. In this experiment, first 10 mL dye of a certain concentration was deposited in a series of Ermalnayer flasks and by adding ZnO-MnFe_2_O_4_/BC sample to it, one experiment parameter was changed while the rest of the parameters were kept fixed. For example, during dose optimization, 10 mL dye of 20 mg/L concentration was deposited in a series of Ermalnayer flasks and several different ZnO-MnFe_2_O_4_/BC doses (1–5 g/L) were added to it and shaken for 100 min with a water bath shaker, 150 RPM at 7 pH. Similarly, many other parameters were also optimized. After a certain time, the dye solution was separated out from the solution mixture (having adsorbent) and then the concentration of dye present in the separated solution (supernatant) was spectrophotometrically {UV-Vis spectrophotometer (T80-UV/VIS, PG Instruments Ltd., Leicestershire, Earl Shilton, UK)} taken out. The adsorption efficiency (removal efficiency (%)) and adsorption capacity (*Q*_e_, mg/g) of the ZnO-MnFe_2_O_4_/BC are calculated from the post-adsorption concentration (*C*_e_, mg/L) and the pre-adsorption concentration (*C*_o_, mg/L) using Equations (1) and (2) [29].

(1) $$Removal\;\left(\%\right)=\left(C_{o}-\frac{C_{e}}{C_{o}}\right)100$$

(2) $$Q_{e}=\left(C_{o}-C_{e}\right)\frac{V}{m}$$

where *V* (L) is the volume of dye solution and *m* (g) is a dose of adsorbent.

It is important to note here that the concentration of the dye in the solution before adsorption was determined spectroscopically, for which a calibration graph was drawn between the concentration of the dye at different concentrations and the absorbance at that concentration using the Beer–Lambert law (Figure S1). The concentration of the post-adsorption dye was determined using the absorbance of solution divided by the slope obtained from the calibration graph. The absorbance value of the dye solution was noted down at λ maximum value of 500 nm [30].

## 3. Result and Discussion

### 3.1. Structural Characterization

Various spectroscopic and microscopic analyses were performed to characterize the resulting nanocomposite.

#### 3.1.1. FTIR Analysis

**Figure 1:**
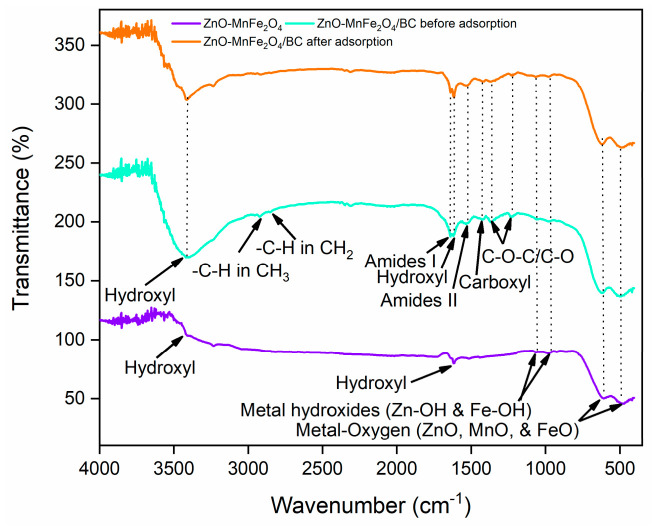
FTIR of ZnO-MnFe_2_O_4_, ZnO-MnFe_2_O_4_/BC before CR adsorption, and ZnO-MnFe_2_O_4_ after CR adsorption.

FTIR analysis was conducted at the 400 to 4000 cm^−1^ wavenumber range (Figure 1). The FTIR analysis clearly revealed the various organic functional groups of the nanocomposite (in comparison to the virgin ZnO-MnFe_2_O_4_) and the interactions between the BC seeds and the ZnO-MnFe_2_O_4_ NPs. The FTIR of the BC seed powder (Figure 1) revealed various functional groups, such as hydroxy, carbonyl, amide I and II, and carboxylic, along with some other organic functional groups, which clearly reflect the cellulosic and protein structure of the BC seeds [23,25,28]. All these peaks are shown in Table 1. These functional groups on the surface of the BC seeds act as interaction sites for the BC and help the NPs grow on the BC surface [26,27]. Therefore, all these functional groups were also found in the FTIR of the current nanocomposite. Apart from these functional groups, metal hydroxide and oxygen particle peaks were also found in the FTIR of the nanocomposite, which represent Zn-O, Fe-O, and Mn-O [31,32]. After the incorporation of the ZnO-MnFe_2_O_4_, a shift was observed in all the peaks of BC seeds, while some disappeared, and the intensity also decreased. This confirms that the interaction between the nanoparticles (ZnO-MnFe_2_O_4_) and the BC surface was due to electrostatic and non-electrostatic interactions, which acted as a strong bond and played a role in the formation of the nanocomposite. Similar findings have been reported in several previous studies [26,29]. All these shifts and peaks are shown in Table 1. Therefore, the FTIR analysis confirms the formation of nanocomposites along with the various functional groups on the nanocomposite surface.

#### 3.1.2. XRD Analysis

**Figure 2:**
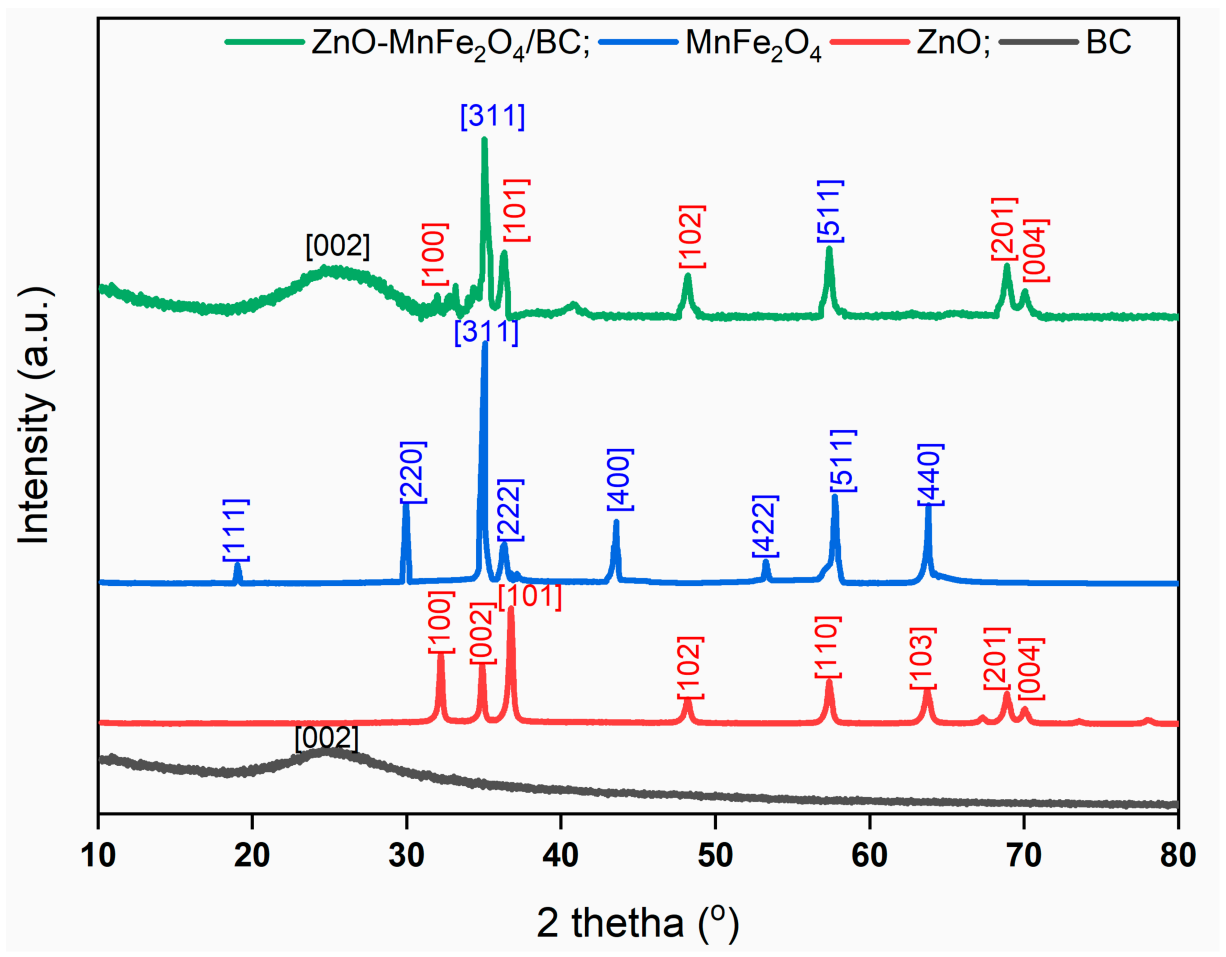
XRD analysis of BC seed powder, ZnO, MnFe_2_O_4_, and ZnO-MnFe_2_O_4_/BC.

X-Ray diffraction analysis was performed to study the phase, plane, and crystal structure of the prepared nanocomposite. The composite’s XRD was compared with that of the BC seed, ZnO, and MnFe_2_O_4_ NPs (Figure 2). It was found that the resulting NPs showed characteristic XRD peak pattern for ZnO {JCPDS Card No: 00-036-1451} [33], and MnFe_2_O_4_ NPs {JCPDS Card No: 10-0319} [34], which were also present in the XRD of prepared nanocomposite [31]. Furthermore, the nanocomposite also showed peaks for carbon of BC seeds at 20 to 25° [25]. The XRD intensities were also slightly reduced and shifted in nanocomposite pattern, indicating the interaction between the BC seed surface and the ZnO-MnFe_2_O_4_ NPs. The Scherer equation (Equation (3)) was used to determine the crystal size of the prepared nanocomposite. Using the Scherer equation, the crystal size of the prepared nanocomposite was determined to be 45.6 nm.

(3) $$D=\frac{K\lambda }{\beta cos\;\theta }$$

where *β* is the full width half maximum, FWHM, *θ* is Bragg’s angle (°) and *λ* is a wavelength of the X-ray radiation (*λ* = 0.154 nm).

#### 3.1.3. SEM and TEM Analysis

**Figure 3:**
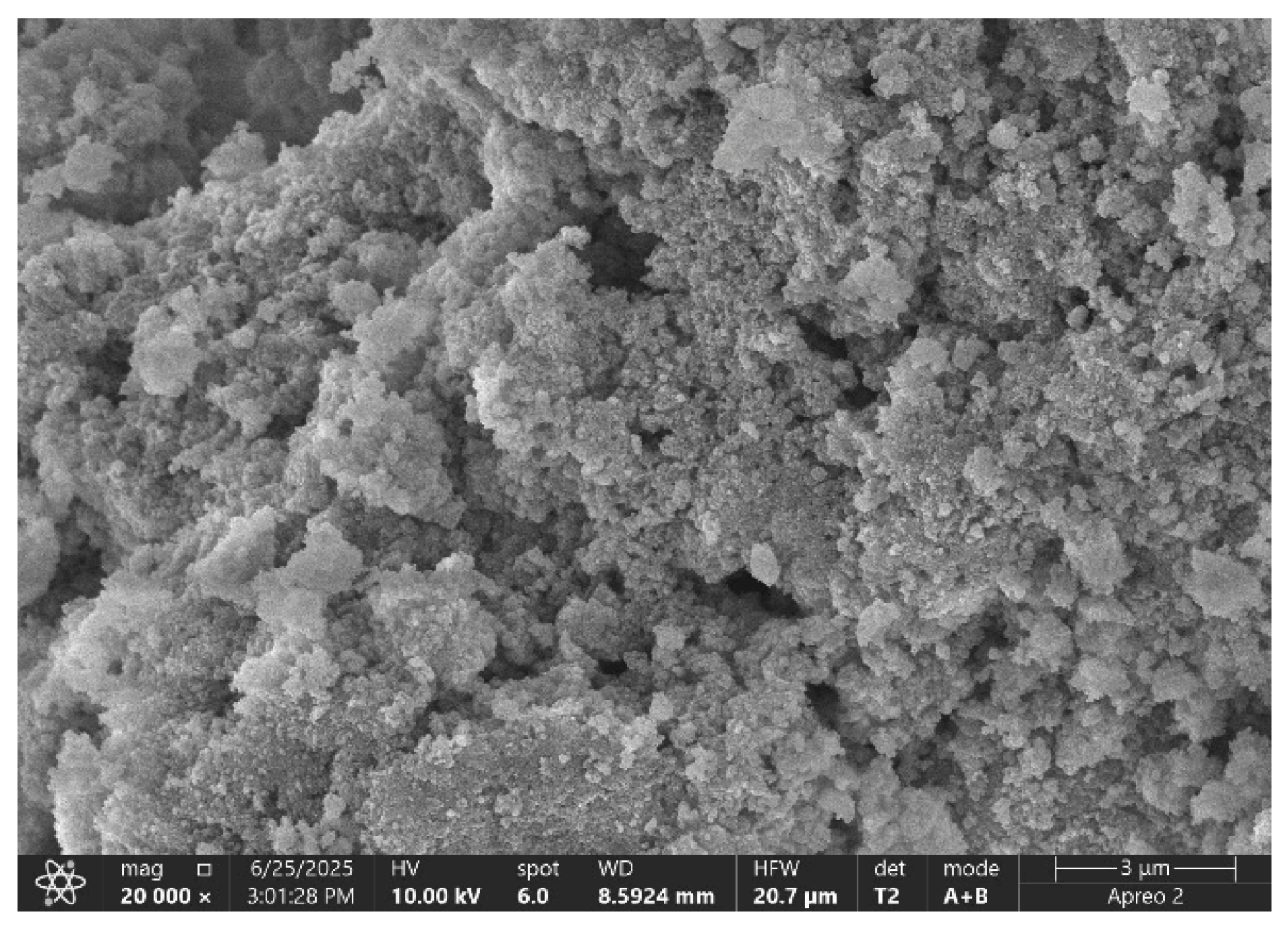

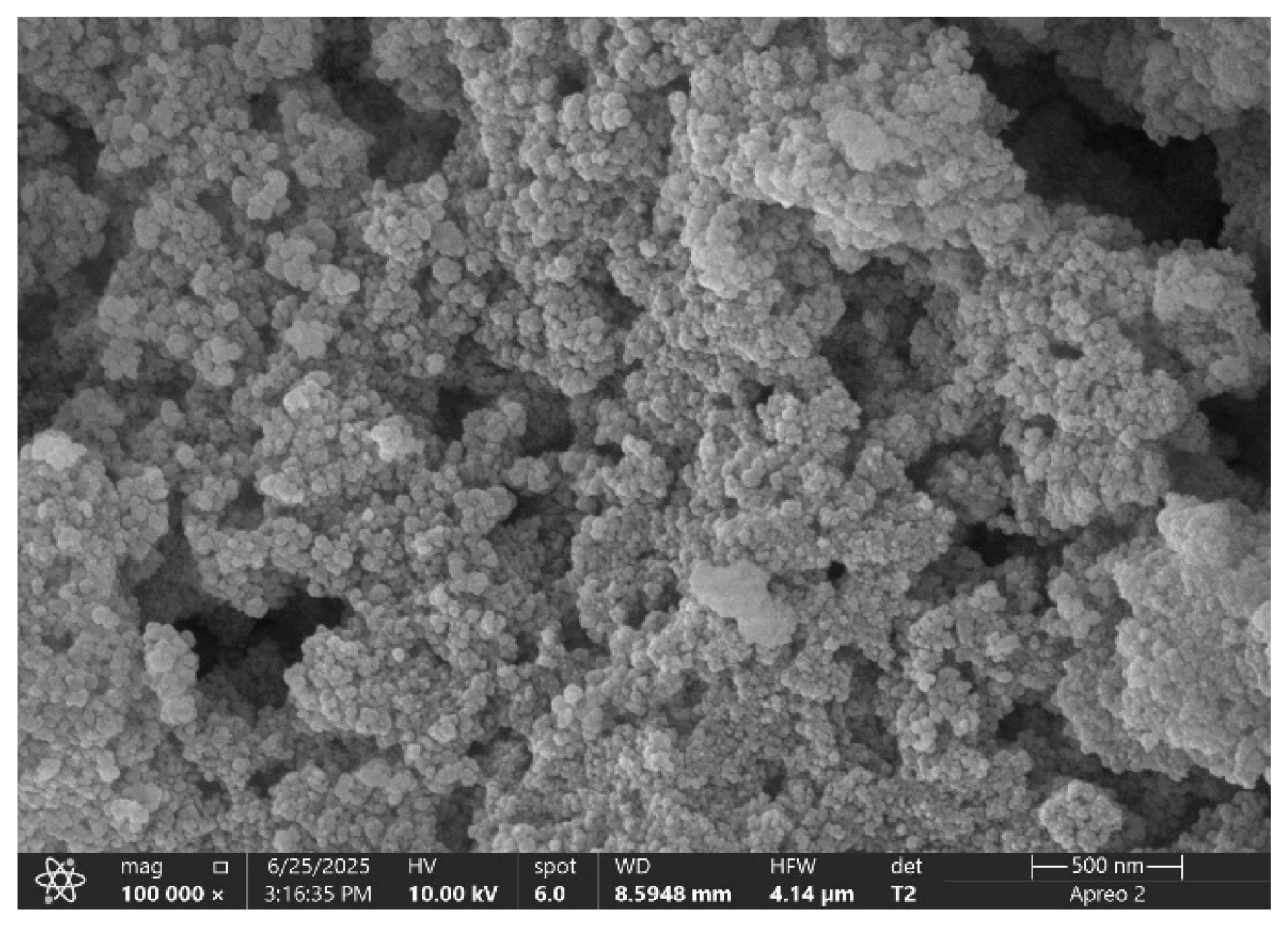
Images of SEM for ZnO-MnFe_2_O_4_/BC.

SEM analysis was also conducted for the prepared nanocomposite, which revealed the surface morphology (Figure 3). The agglomerated nanoparticle system can also be observed from the SEM images. Upon examining the surface morphology, it was found that the surface of the nanocomposite consists of irregular clusters of nanoparticles which forms the porous and sponge-like morphology. The voids and interparticle pores can also be observed in the SEM images. This kind of morphology suggested the carbon-based nanostructures.

**Figure 4:**
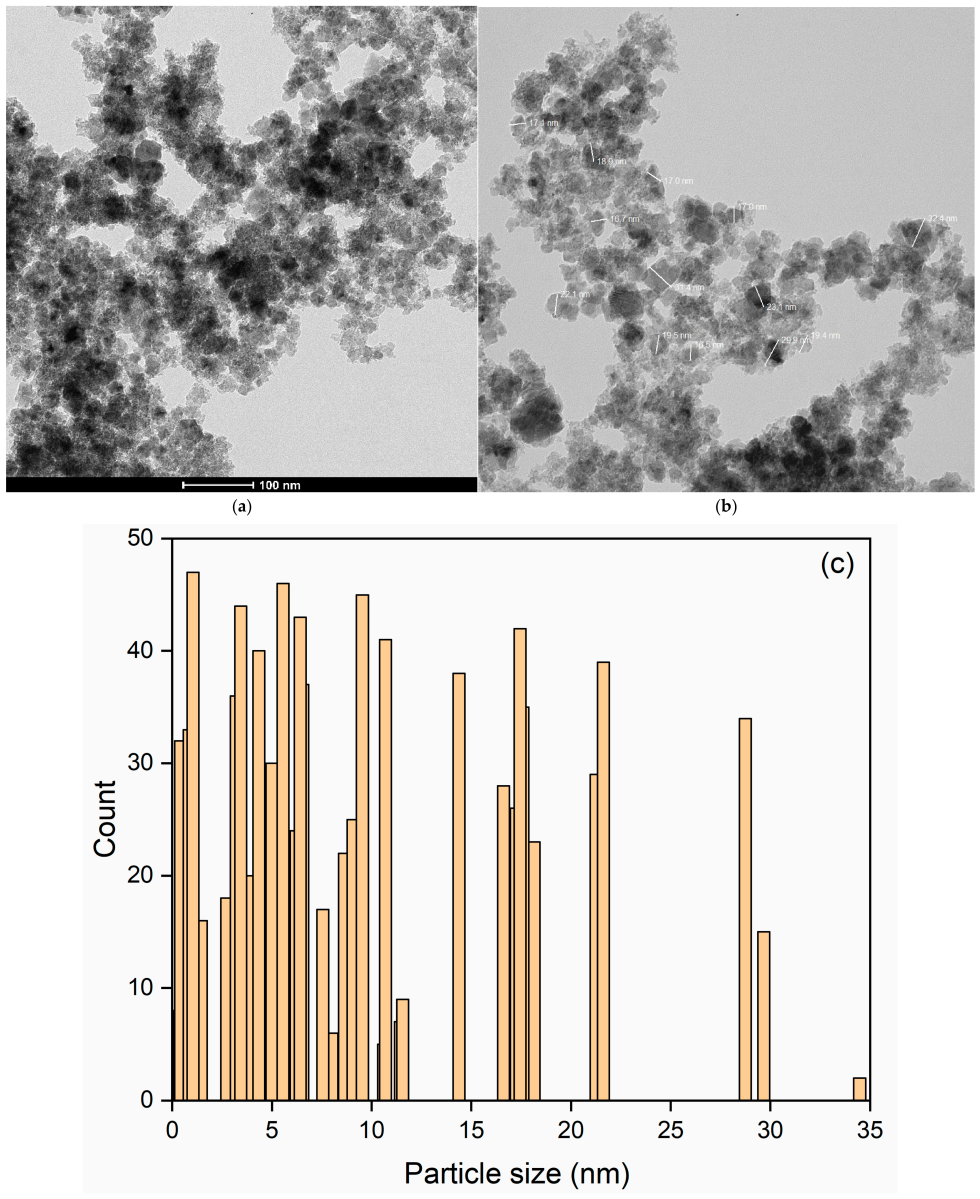
(**a**,**b**) TEM images of ZnO-MnFe_2_O_4_/BC and (**c**) particle size distribution histogram.

The TEM images also give the same observation (highly agglomerated, the porous and sponge-like carbon-based nanostructure) (Figure 4). The dark region shows the aggregated nanoparticles while the bright region reveals the voids and interparticle pores (Figure 4). The TEM image particle size was found to be in the range ~15–35 nm.

The particle size distribution histogramc) reveals that the synthesized nanocomposite consists of nano-ranged particles with moderate size variation. The particles concentrated around the diameter of 2–12 nm. A small extent of large particles (extended up to ~35 nm) suggests the aggregation of nanoparticles.

#### 3.1.4. EDAX Analysis

**Figure 5:**
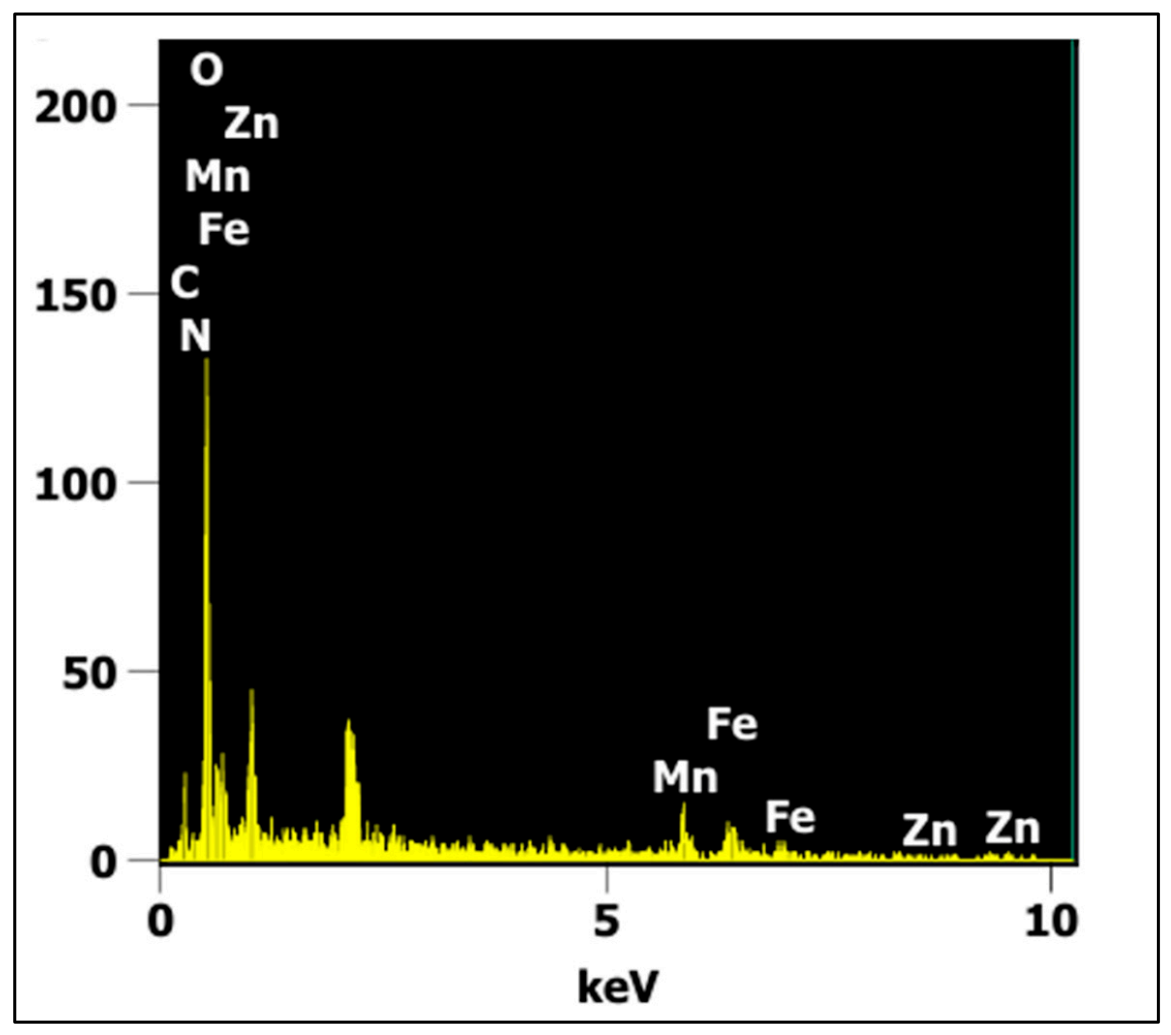
Results of EDX analysis for ZnO-MnFe_2_O_4_/BC.

Element analysis revealed O, Zn, Mn, and Fe presence in the nanocomposite (Figure 5), which was due to the presence of ZnO-MnFe_2_O_4_ NPs. In addition, C, N, and additional O were also found in the nanocomposite, which was due to the BC seed framework. Through element mapping (Figure S2), it can be seen that C had the highest distribution in the nanocomposite. Apart from that, Zn, Fe, and Mn also showed a uniform distribution. This analysis also confirms that the ZnO-MnFe_2_O_4_ NPs are present in the formed nanocomposite along with the high content of C.

#### 3.1.5. TG Analysis

TG analysis of the prepared nanocomposite was also carried out. TGA showed that the present nanocomposite remains quite stable up to 850 °C (Figure S3). This can be briefly explained as follows: first a decrease was observed at 110 °C, indicating the removal of highly volatile compounds like moisture. After that, a second decrease was observed at 120–350 °C, indicating the destruction of oil present on the BC surface. After that, a continuous decrease was observed from 350 to ~600 °C, indicating pyrolysis, which leads to the formation of carbon residues. After that, a steady-state decrease was observed up to 880 °C, indicating the removal of carbon residues as oxides. Approximately 86% of the nanocomposite sample mass was retained after the 850 °C of calcination, suggesting a high stability of the prepared nanocomposite [29].

By comparing all these spectroscopic and microscopic analyses of the present sample with several previous reported nanocomposites, it can be clearly revealed that in the current sample preparation, there is a strong interaction between the BC seed surface and ZnO-MnFe_2_O_4_ NPs (confirmed by FTIR, XRD, SEM, and EDX), confirming the formation of novel nanocomposite.

### 3.2. Adsorption Study

Adsorption analysis was explained through a batch adsorption experiment in which adsorption performance was determined under several variable conditions as reported below.

For dose optimization, five doses of 1, 2, 3, 4, and 5 g/L of ZnO-MnFe_2_O_4_/BC sample were taken and mixed with 10 mL of 10 mg/L of CR dye. The mixture was then shaken by a water bath shaker at 25 °C, 7 pH, and 150 RPM speed for 90 min. The adsorption result (Figure S4a) showed that as the adsorbent dose increases, the adsorption efficiency increases and an equilibrium is reached after a dose of 2 mg/g adsorbent sample. As is clear from the formula itself, adsorbent dose is directly proportional to the removal efficiency. As the dose increases, the active site concentration on the adsorbent surface also increases which leads to an increase in adsorption efficiency [29]. At 1 g/L only 47% dye was removed whereas at 2 g/L, almost 100% dye was removed. Since a limited concentration of dye is supplied (i.e., 10 mg/L), at the 2 g/L dose of adsorbent almost all the dye (~100%) was removed and there was no dye left in the solution; thus equilibrium was achieved. Therefore, for subsequent analysis the concentration of dye was increased. At 2 g/L, dye adsorption capacity (Q_e_, mg/g) was reported at about ~5.0 mg/g, which proved to be better than many previous studies [29,35].

This dose (i.e., 2 g/L) was further used to optimize the concentration of dye under similar conditions for other variables such as volume, pH, time, temperature, and RPM. It was observed that 2 g/L was able to remove about ~45% of dye even from a 10 mL of 50 mg/L solution (Figure S4b). This dose of adsorbent was able to remove about ~82% and ~63% of dye at concentrations of 20 and 30 mg/L, respectively, which clearly shows that the present adsorbent was significant for the present dye adsorption. With 2 g/L, *Q*_e_ was found to be 11.3 mg/g at 50 mg/L CR concentration.

In similar experimental conditions (adsorbent dose: 2 g/L; pH: 7; concentration: 10 mg/L; and RPM: 150), the temperature was increased (25 to 55 °C) and it was observed that the adsorption efficiency and capacity decreased with increasing temperature (Figure S4c). This indicates an exothermic process.

The presence of various pollutants in natural water sources affects the pH of the water. This altered pH also affects the dye adsorption capacity of adsorbents containing functional groups. Since the adsorbent used in the current study was highly functionalized, the charge specificity of these functional groups varies with pHs. For example, at low (acidic) pH, protonation occurs on the adsorbent surface, making it positive, whereas at higher (basic) pH, deprotonation increases the negative charge on the adsorbent surface [27].

The current adsorption process used CR dye, which is an anionic dye. Therefore, this dye is more readily removed by the ZnO-MnFe_2_O_4_/BC adsorbent at lower pH, while at basic pH, the adsorption capacity of the adsorbent for CR decreases significantly. The effect of pH for the current study is shown in Figure S4d [36].

For kinetic study, the removal efficiency was determined by varying the time at optimized conditions. Figure S4e shows the adsorption efficiency at different times and it can be determined that initially the adsorption is very fast (in the first 30 min) and it becomes very slow later on. About 71% of the dye is removed in the first 30 min while it takes about 90 min more for the remaining 27% of dye to be removed. This indicates that initially, the adsorption sites were completely unoccupied, so the solute (dye molecules) does not require a specific adsorption site; instead, the mass transfer occurs, resulting in rapid adsorption onto the nanocomposite surface. However, as the time progresses, the solute requires a specific site for adsorption, requiring some assistance. Thus, the adsorption rate decreases. After 90 min, a steady state is reached, indicating equilibrium. By 90 min of adsorption process, almost 100% of the dye had been removed [29].

### 3.3. Thermal Analysis

The obtained temperature and concentration experimental adsorption data were used for thermodynamic and isothermal analysis.

#### 3.3.1. Thermodynamics Analysis

Van’t Hoff equation was used for thermodynamic analysis (Equation (4)) [37]:

(4) ∆G° (kJ/mol)= ∆H° − T∆S°

where ∆*G*° (kJ/mol), ∆*H*° (kJ/mol), and ∆*S*° (kJ/mol/K) are the thermodynamic parameters which represent free energy change, enthalpy change, and entropy change, respectively.

A slope and intercept of graph plotting ∆*G*° (calculated by Equation (5)) versus *T* (reaction temperature in K) gives the values of ∆*H*° and ∆*S°*.

(5) ∆G° (kJ/mol)=−RTlnKc

where R is the universal gas constant (R = 8.3145 J/mol K) and Kc is the equilibrium constant (Kc = *Q*_e_/*C*_e_).

The negative ∆*G*° values indicate spontaneity and feasibly, while a negative ∆*H*° value indicates the exothermic nature of the adsorption process and a positive value indicates endothermic nature. As the value of ∆*S*° increases, the randomness in the system increases, i.e., a positive value indicates randomness, while a negative value indicates a decrease in randomness [38,39].

For the current study, the thermodynamic analysis revealed negative ∆*G*° values at all temperatures (Figure S4f; Table 1), indicating that the adsorption process is feasible and spontaneous. The values of ∆*H*° and ∆*S*° were found to be negative (Table 1), indicating an exothermic process and reduction in randomness at the solid–liquid interface, respectively [37].

#### 3.3.2. Isotherm Analysis

**Figure 6:**
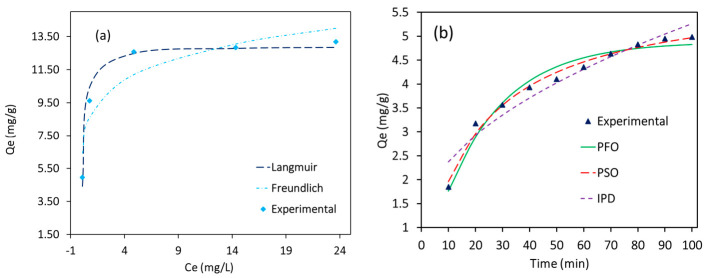
Non-linear plot of (**a**) isotherms and (**b**) kinetics for CR adsorption onto the ZnO-MnFe_2_O_4_/BC (experimental conditions: Adsorbent dose: 2 g/L; concentration: 10 mg/L; time: 10–100 min; temperature: 25 °C; pH: 7; and shaker speed: 150 RPM).

Two non-linear isotherm models (Langmuir and Freundlich) were used for the isothermal analysis (Figure 6a; Table 2). The Freundlich model represents heterogeneous and multi-layer adsorption, suggesting physical adsorption, while the Larepresentsdel represent homogeneous and monolayer adsorption, assuming chemical adsorption. These two models can be expressed using non-linear Equations (6) (for Langmuir model) and (7) (for Freundlich model) [27,29].

(6) $$Q_{e}(mg/g)=\frac{Q_{o}bC_{e}}{1+bC_{e}}$$

(7) $$Q_{e}\;(mg/g)=kFC_{e}^{1/n}$$

where *Q*_o_ is the maximum adsorption capacity in mg/g, b (L/mg) is a Langmuir constant refering to adsorption intensity, *n* is a heterogeneity (confirmed by the value ranging from 1 to 10), and kF ([(mg/g)(L/mg)^1/*n*^]) is the Freundlich isotherm constant, representing Freundlich adsorption capacity.

It was observed that the error functions for the Langmuir model were lower than the Freundlich model, suggesting a good fit of the Langmuir model. This fitting can also be confirmed by comparing the theoretical adsorption data to experimental adsorption data (Table S1), suggesting high agreement with the Langmuir model.

This result suggests that there was a monolayer and chemical adsorption on the ZnO-MnFe_2_O_4_/BC surface. In thermodynamic analysis, the value of ∆*H* negative was found to be more than 40 kJ/mol which also confirms the chemical adsorption. With this result it can be confirmed that chemical adsorption mechanism was effective in the present adsorption process.

### 3.4. Kinetic Study

To understand the kinetics, a non-linear plot of a pseudo-first-order (PFO) (represented by Equation (8)), pseudo-second-order (PSO) (represented by Equation (9)), and Weber–Morris (WM) (represented by Equation (10)) model were applied (Figure 6b; Table 3). The PFO plot represents physical adsorption (assuming only the adsorbent surface is responsible for adsorption), while the PSO plot represents chemical adsorption (assuming that both the adsorbent surface and pollutant nature are responsible for adsorption). The WM plot represents a rate-determining (slow) step [39] by assuming that adsorption is a multi-step process, governed through film (boundary layer) diffusion or intraparticle diffusion.

(8) $$Q_{t}\;(mg/g)=q_{e}(1-e^{-k_{1}t})$$

(9) $$Q_{t}(mg/g)=\frac{Q_{e}^{2}K_{2}t}{1+Q_{e}K_{2}t}$$

(10) $$Q_{t}(mg/g)={k_{\mathrm{ipd}}}^{0.5}+C$$

Herein, *Q*_t_ (mg/g) and *Q*_e_ (mg/g) are adsorption capacity at time t and equilibrium, and K_1_, K_2_, and K_ipd_ are rate constants for PFO, PSO, and WM kinetics, respectively. C (the intercept of WM plot) represents the boundary layer thickness.

It can be seen from the current non-linear plots that the PSO kinetic shows better agreement to the experimental results (with lowest error functions, χ^2^ and SSR) than the PFO kinetic (Table S2), meaning that chemical adsorption predominantly determines the current adsorption.

The WM model was used to interpret the rate-determining step. In this graph, the value of C (intercept) was not found to be zero (did not pass through the origin), which indicates that the current adsorption process was multi-step, i.e., interparticle diffusion and boundary layer (film diffusion) were both deciding factors in the current adsorption mechanism. However, film diffusion (chemical adsorption) contributed more to the current adsorption process as the value of C was found to be far away from zero (i.e., 1.04).

### 3.5. Regeneration and Reusability Test

The stability of the existing adsorbent, i.e., regeneration and reused power, was also observed. The regeneration process was carried out by adding exhausted adsorbent to 0.1 molar NaOH solution. For regeneration, 1 g of adsorbent collected post-adsorption was dispersed in 100 mL solution of 0.1 molar NaOH for 300 min. After regenerating, adsorbent was thoroughly washed with distilled water, and then dried. The adsorption performance of regenerated adsorbent was observed by experimenting with the adsorption process, taking 2 g/L regenerated adsorbent under the optimized conditions. The adsorption efficiency was reduced by ~25% after the second cycle (Figure S5). This process was repeated for five reused cycles and it was found that after the fifth cycle, the adsorption removal efficiency reduced by ~65%.

Although the removal efficiency remained quite low until the fifth cycle, this might be mainly due to the loss of a certain amount of adsorbent after each adsorption cycle, leading to a decrease in its overall mass. Additionally, it is possible that the adsorption sites may have been exhausted due to chemical interactions. Furthermore, some surface damage or agglomeration of particles may occur after the adsorption process, which could also contribute to the reduced adsorption efficiency. These are all possible reasons that might be responsible for this reduced adsorption efficiency.

### 3.6. Comparative Study

The adsorption data (capacity and efficiency) obtained in this study was compared, along with partition coefficient (PC) values, with the previous adsorption data reported in the literature under various conditions. Notably, the PC is the less biased comparative parameter under the various operational conditions. It can be calculated by Equation (11) [40,41].

(11) $$PC=Adsorption\;capacity\;(Q_{e})/Initial\;concentration\;(C_{o})*removal\;rate\;(Q_{t})$$

It was found that the current adsorbent showed better performance than some of the previously reported adsorbents as shown in Table 4.

## 4. Conclusions

In this study, a nanocomposite was prepared by growing zinc oxide-manganese ferrite on black cumin seed surface, which served as a carbon framework. This nanocomposite was used to remove Congo red dye from water. Due to the large functional groups, the resulting composite was highly effective in removing Congo red dye. A two-gram-per-liter amount of the resulting nanocomposite removed nearly 100% of the dye from a solution with a concentration of 10 milli gram per liter at pH 7 in 90 min. The adsorption process was exothermic, physicochemical, and followed the Freundlich isotherm, pseudo-second-order kinetics, and the mechanism was described by film diffusion and intraparticle diffusion. The resulting nanocomposite was reused for five cycles, and it was found that the nanocomposite performed significantly better in the first two cycles. This nanocomposite demonstrates the potential of nanocomposites made with biomaterials in wastewater treatment and also suggests the possibility of photodegradation using these materials in the future.

## Figures and Tables

**Table 1:** Thermodynamic parameters for CR adsorption onto the ZnO-MnFe_2_O_4_/BC (experimental conditions: Adsorbent dose: 2.0 g/L; concentration: 10 mg/L; time: 90 min; temperature: 25–55 °C; pH: 7; and shaker speed: 150 RPM).

Temperature (K)	∆*G*° (kJ/mol)	∆*S*° (kJ/mol/K)	∆*H*° (kJ/mol)
298	−9.821	−0.266	−89.152
308	−6.984		
318	−4.235		
328	−1.853		

**Table 2:** Result of various isotherm parameters for CR adsorption onto the ZnO-MnFe_2_O_4_/BC (experimental conditions: Adsorbent dose: 2 g/L; concentration: 10–50 mg/L; time: 90 min; temperature: 25 °C; pH: 7; and shaker speed: 150 RPM).

Model	Langmuir				Freundlich			
Parameters	Q_max_ (mg/g)	b (L/mg)	χ^2^	SSR	*n*	kF ([(mg/g)(L/mg)^1/*n*^])	χ^2^	SSR
Results	12.95	5.51	0.020	1.06	7.10	9.0	0.11	5.80

**Table 3:** Kinetics parameters for CR adsorption onto the ZnO-MnFe_2_O_4_/BC (experimental conditions: Adsorbent dose: 2 g/L; concentration: 10 mg/L; time: 10–100 min; temperature: 25 °C; pH: 7; and shaker speed: 150 RPM).

Kinetic Model	Parameters	Values
PFO	K_1_ (min^−1^)	0.045
	*Q*_e_ (mg/g)	4.90
	SSR	0.275
	χ^2^	0.007
PSO	K_2_ (min^−1^)	0.008
	*Q*_e_ (mg/g)	6.00
	SSR	0.106
	χ^2^	0.002
WM	K_ipd_ (mg/g/min^0.5^)	0.422
	C	1.040
	SSR	0.534
	χ^2^	0.013

**Table 4:** Comparative study of ZnO-MnFe_2_O_4_/BC with other reported adsorbents under various conditions. Adapted from [29,35] (reprinted with permission from Fatima et al. (2021a), Copyright (2021) Elsevier).

Order	Adsorbents	Initial Concentration of CR (mg/L)	Time (min)	Dose (g/L)	Temp. (°C)	pH	*Q*_e_ (mg/g)	Removal (%)	*Q*_o_ (mg/g)	PC (mg/g/μM)	Ref.
1.	Modified bentonite	20.00	60	4.00	25	8.00	4.80	96.40	22.30	4.70	[29]
2.	SiO_2_/C	100.0	2160	0.10	30	7.00	957.0	95.70	2512.0	155.0	
3.	RF carbon gel	10.00	7200	1.50	-	5.40	3.30	49.80	10.60	0.46	
4.	ZnFe_2_O_4_/SiO_2_/TG	10.00	60	0.20	-	5.00	22.50	50.00	128.2	3.10	
5.	ZnO-CdWO_4_	10.00	60	2.00	30	7.00	5.00	98.40	26.50	21.40	
6.	CuO-ZnO-CdWO_4_	20.00	135	2.50	30	7.00	3.98	99.66	19.64	83.32	[38]
7.	ZnO-MnFe_2_O_4_/BC	10.00	90	2.00	25	7.00	4.95	99.06	12.95	36.88	This Study

## Data Availability

The data that support the findings of this study are available from the corresponding authors, upon reasonable request.

## Supplementary Materials

The following supporting information can be downloaded at: https://www.mdpi.com/article/10.3390/nano16120775/s1, Figure S1: Calibration curve for CR dye; Figure S2: Elemental mapping of ZnO-MnFe_2_O_4_/BC, Figure S3: TG analysis result of ZnO-MnFe_2_O_4_/BC, Figure S4. Plots of optimization of various parameters such as (a) adsorbent dose, (b) concentration, (c) temperature, (d) pH, (e) contact time, and (f) thermodynamic plot for CR adsorption on to ZnO-MnFe_2_O_4_/BC, Figure S5. Regeneration and reusability results of ZnO-MnFe_2_O_4_/BC for CR adsorption Table S1: Comparative analysis of experimental and theoretical (using isotherms model) adsorption capacities at various concentration; Table S2. Comparative analysis of experimental and theoretical (using kinetics model) adsorption capacities at various interval time (Experimental conditions: Temperature = 25 °C; pH = 7.0; Adsorbent dose = 2.0 g/L; Concentration = 10 mg/L; Agitation speed = 200 RPM).
