# Supplementary material for: Removal of Congo Red Dye from Aqueous Solution via Natural Seeds Integrated with Zinc Oxide-Doped Manganese Ferrite
Source: Nanomaterials (Basel). 2026 Jun 19;16(12):775. doi: 10.3390/nano16120775 (PMC13305837; doi:10.3390/nano16120775)
Supplement: Supplementary file 1 [file nanomaterials-16-00775-s001.zip › nanomaterials-4315630-supplementary.pdf]

Electronic supporting information

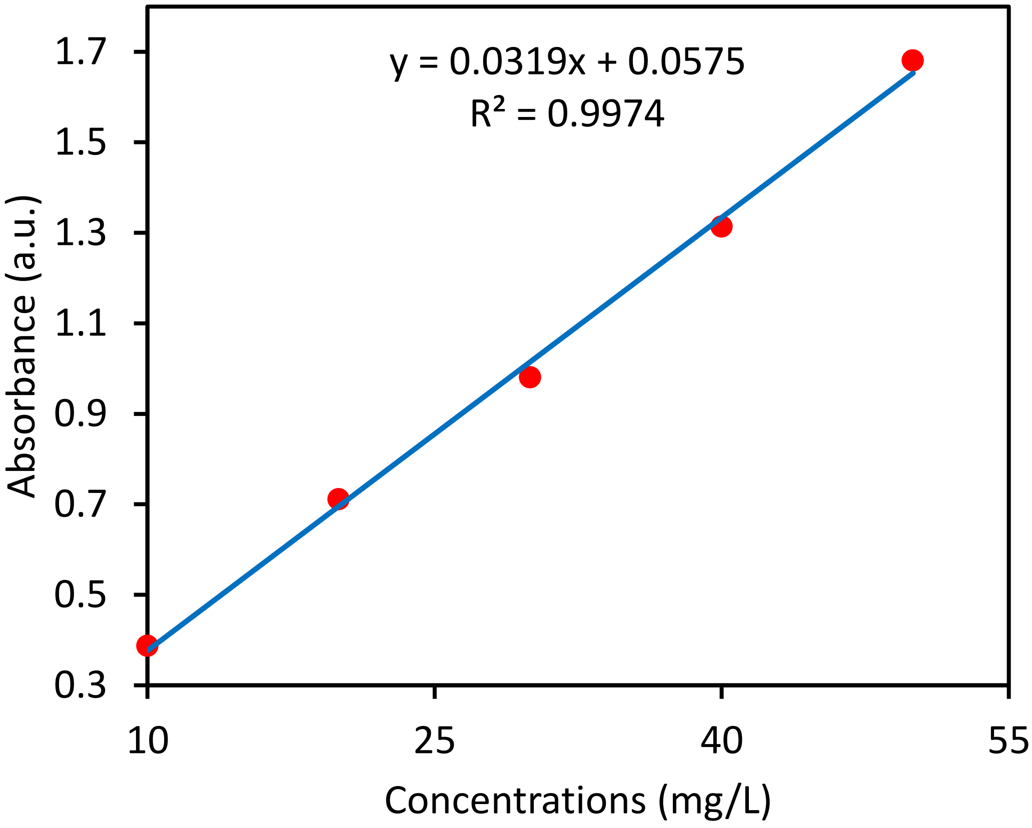

Figure S1. Calibration curve for CR dye

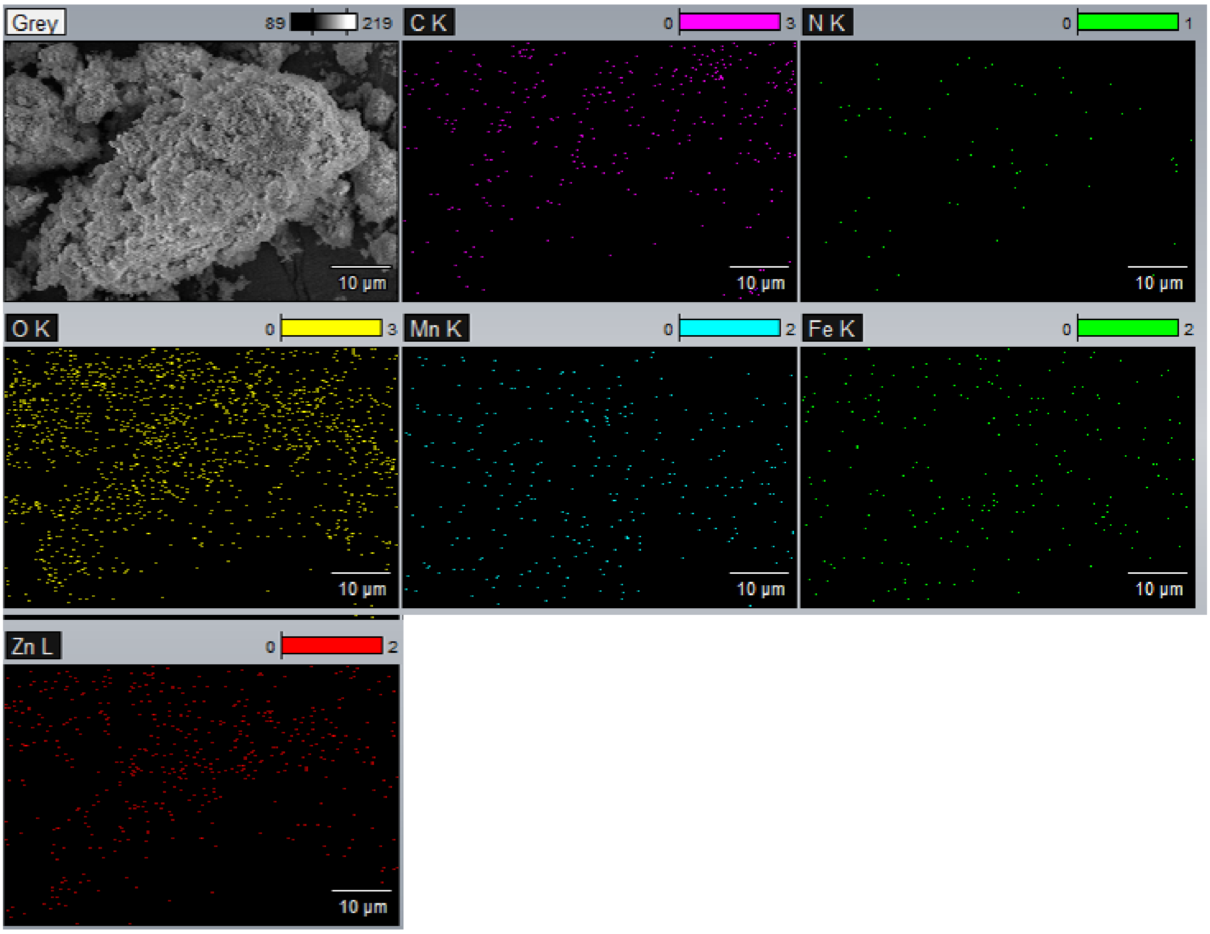

Figure S2. Elemental mapping of ZnO-$MnFe_2O_4$/BC

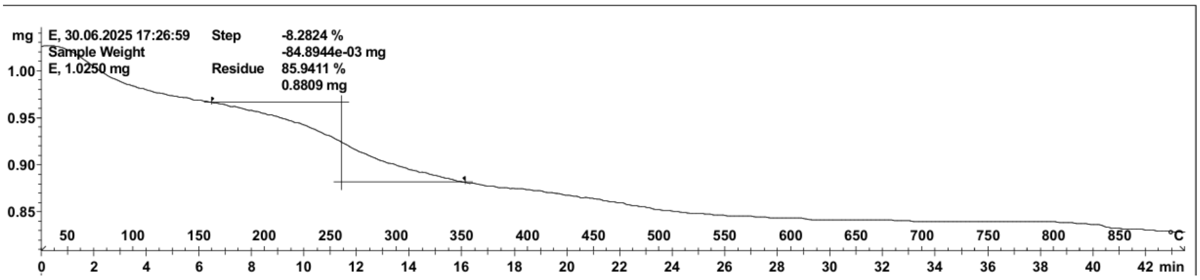

Figure S3. TG analysis result of ZnO-$MnFe_2O_4$/BC

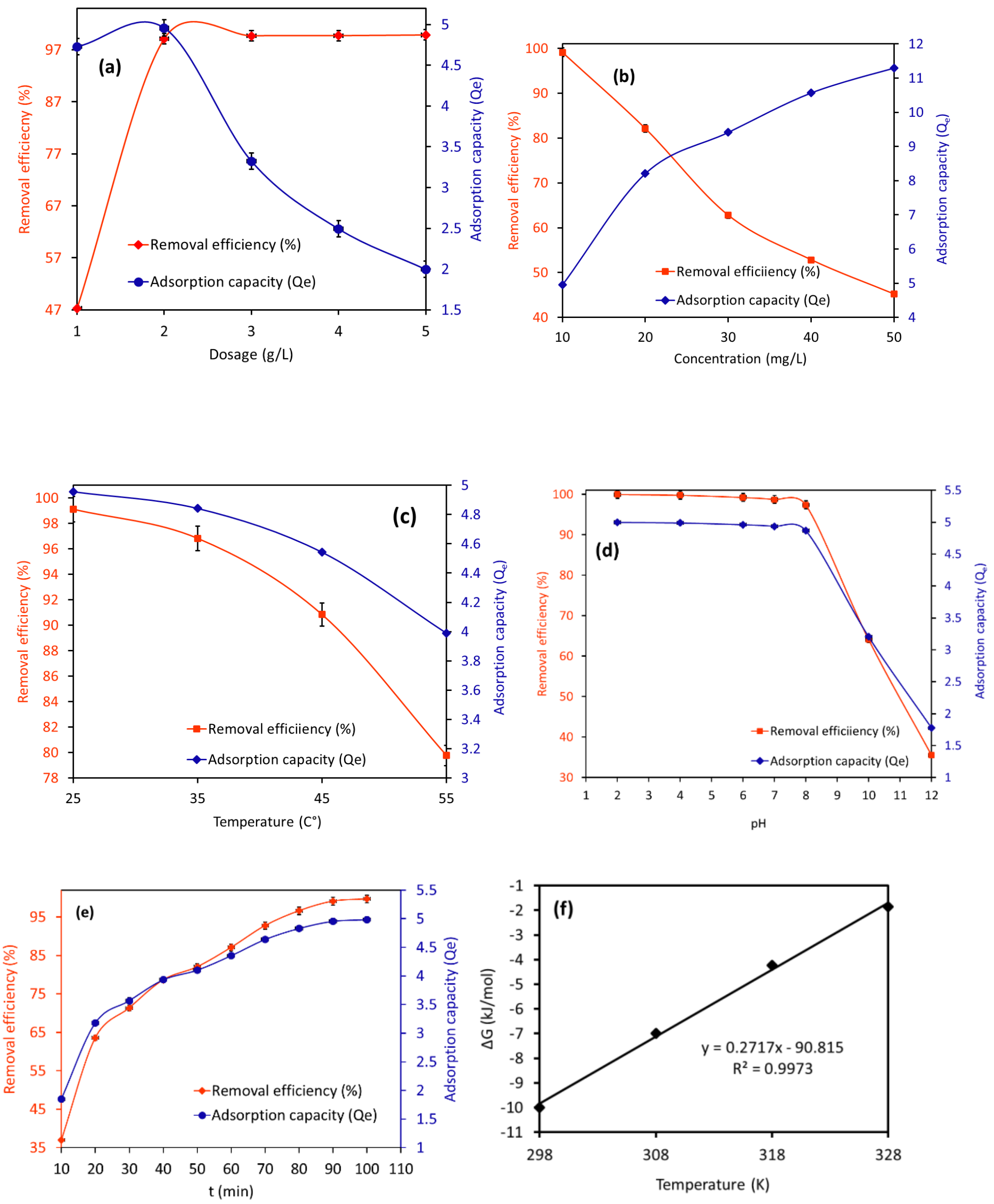

Figure S4. Plots of optimization of various parameters such as (a) adsorbent dose, (b) concentration, (c) temperature, (d) pH, (e) contact time, and (f) thermodynamic plot for CR adsorption on to ZnO-$MnFe_2O_4$/BC.

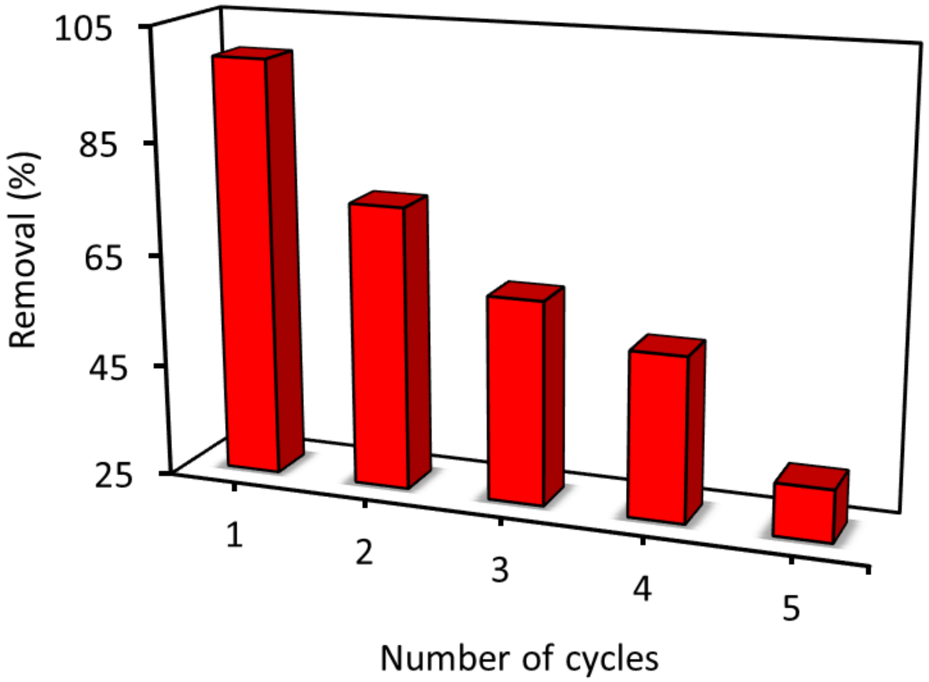

Figure S5. Regeneration and reusability results of $ZnO-MnFe_2O_4/BC$ for CR adsorption

Table S1. Comparative analysis of experimental and theoretical (using isotherms model) adsorption capacities at various concentration (Experimental conditions: Temperature = 25 °C; pH = 7.0; Adsorbent dose = 2.0 g/L; Contact time = 90 min; Agitation speed = 200 RPM).

Concentration (mg/L)	**Adsorption capacity (Qe)**		
	Experimental	Langmuir	Freundlich
10	4.95	4.42	6.43
20	9.62	10.43	8.62
30	12.57	12.48	11.21
40	12.84	12.79	13.05
50	13.18	12.85	14.01

Table S2. Comparative analysis of experimental and theoretical (using kinetics model) adsorption capacities at various interval time (Experimental conditions: Temperature = 25 °C; pH = 7.0; Adsorbent dose = 2.0 g/L; Concentration = 10 mg/L; Agitation speed = 200 RPM).

Time (min)	**Adsorption capacity (Q_t)**			
	Experimental	PFO	PSO	IPD
10	1.84	1.76	1.96	2.37
20	3.17	2.88	2.95	2.92
30	3.56	3.60	3.55	3.35
40	3.93	4.06	3.95	3.70
50	4.10	4.36	4.24	4.02
60	4.35	4.55	4.46	4.30
70	4.63	4.67	4.63	4.57
80	4.83	4.74	4.76	4.81
90	4.95	4.79	4.87	5.04
100	4.98	4.82	4.97	5.26
